# Analysis of the production in a connected speech task in patients with language impairment

**DOI:** 10.1002/alz.092274

**Published:** 2025-01-03

**Authors:** Santiago Redondo, Patricia Paolantonio, Sofía Romanelli, Daniela Andreotti, Lourdes Matar, Luciana Vita, Leticia Vivas

**Affiliations:** ^1^ Cordoba´s National University, Cordoba, Córdoba Argentina; ^2^ CONICET, Córdoba, Córdoba Argentina; ^3^ Córdoba´s National University, Córdoba Argentina; ^4^ Facultad de Humanidades, UNMDP, Mar del Plata, Mar del Plata Argentina; ^5^ San Isidro´s Hospital, San Isidro Argentina; ^6^ Private Clinic, Mar del Plata Argentina; ^7^ Instituto de Investigaciones en Psicología Básica y Aplicada (IIPBA), Facultad de Filosofía y Humanidades, Universidad Católica de Cuyo., San Juan Argentina; ^8^ ReDLat, San Juan Argentina; ^9^ IPSIBAT (CONICET/National University of Mar del Plata), Mar del Plata, Buenos Aires Argentina

## Abstract

**Background:**

Neuropsychological language assessment batteries usually include connected speech tasks (e.g. the description of a picture). These tasks are assessed using different indicators that cover various levels of language: phonetic and phonological, lexico‐semantic, syntactic and discourse. Objective: to analyze linguistic and acoustic indicators for the picture description task of the Minlinguistic State Examination (MLSE) in its Argentinean version.

**Method:**

The sample included 22 patients with aphasia due to focal and degenerative etiologies (10 APP, 1 DCB, 11 post‐stroke aphasia) classified into three clinical profiles: fluent, anomic and non‐fluent (according to Grossman, 2018) and 23 healthy participants. The task consisted of an oral description of the MLSE picture for 1 minute and it was audio recorded. Acoustic indicators were analyzed using the Praat software.

**Result:**

Regarding acoustic indicators, significant differences were observed in the duration of pauses between the three clinical groups and the control group, while the differences in vocalization and silence segments were significant between the non‐fluent and the control group. Regarding linguistic indicators, a significant difference was observed between the clinical and control groups in the number of nouns, words/time ratio, number of semantic units, the density of semantic units, speaking average, and number of complete utterances; while significant differences were observed between the control group and the non‐fluent and anomic groups in number of adverbs, non‐noun words, closed words, number of verbs, average number of nouns, utterances higher and lower number of words, mean length of utterance, and utterance rate. Besides, significant differences were observed between the fluent aphasia and the control groups in semantic errors and density of ideas; and between the no fluent aphasia and the control groups in number of total utterances and index of discourse. Among the clinical groups, significant differences were observed between the non‐fluent and fluent groups in the number of semantic errors; and between the anomic and non‐fluent groups in the noun/number of words relationship.

**Conclusion:**

These results illustrate how the use of linguistic and acoustic indicators in a connected speech task can provide additional information to identify the type of difficulties presented by aphasia patients.

